# Optic nerve injury models under varying forces

**DOI:** 10.1007/s10792-022-02476-2

**Published:** 2022-08-29

**Authors:** Wu Sun, Guojun Chao, Mengqiu Shang, Qiong Wu, Yanting Xia, Qiping Wei, Jian Zhou, Liang Liao

**Affiliations:** 1 Beijing University of Chinese Medicine, Beijing, China; 2 Eye Hospital Chinese Academy of Chinese Medical Sciences, Beijing, China; 3 Beijing Tongren Hospital, Beijing, China; 4 Dongfang Hospital Beijing University of Chinese Medicine, Beijing, China; 5 Department of Ophthalmology, Dongfang Hospital, Beijing University of Chinese Medicine, Beijing, 100078 China; 6 No. 6, District 1, Fangxing Garden, Fangzhuang, Fengtai District, Beijing, 100078 China

**Keywords:** Optic nerve crush model, Varying forces, Lateral pulling optic nerve model, Retinal ganglion cells, Flash visual evoked potential

## Abstract

**Purpose:**

To explore the pathological changes in optic nerve injury models under varying forces.

**Methods:**

The rats were classified into 4 groups: sham operation (SH), 0.1, 0.3, and 0.5 N. Modeling was performed using the lateral optic nerve pulling method. Seven days after modeling, Brn3a immunofluorescence was used to detect retinal ganglion cell (RGC) number, terminal deoxynucleotidyl transferase dUTP nick end labeling (TUNEL) staining was used to detect RGC apoptosis, and flash visual evoked potential (FVEP) was used to detect the optic nerve function on days 1, 3, and 7 after modeling. In addition, LC3 II and P62 expression levels in retinal tissues were detected by western blotting to observe the changes in autophagy levels.

**Results:**

RGC number decreased 7 d after modeling, and it showed a downward trend with increasing damaging force. The number of apoptotic RGCs in ganglion cell layer in the 0.3 and 0.5 N groups was increased and was higher than that in the 0.1 N group. The difference in FVEP of rats in each group was mainly reflected in the P2 peak latency. LC3 II and P62 expression levels in retinal tissue of 0.3 and 0.5 N groups were higher than those of the SH and 0.1 groups; however, the difference between the 0.1 N and SH groups was not statistically significant.

**Conclusion:**

Precisely controlling the force of the optic nerve clamping injury model is necessary because different forces acting on the optic nerve will lead to differences in the loss of optic neurons, the conduction function of the optic nerve, and autophagy level in retinal tissues.

**Supplementary Information:**

The online version contains supplementary material available at 10.1007/s10792-022-02476-2.

## Introduction

The optic nerve crush (ONC) is a commonly used model to study optic nerve injury and glaucoma in addition to the survival and regeneration of retinal ganglion cells (RGCs) [1, 2] and the effects of drug or other therapeutic interventions on RGCs [3–8]. It provides good repeatability and simple operation. However, the degree of damage of this model cannot be precisely controlled, leading to bias in the experimental results [9].

The lateral pulling optic nerve (LPON) model quantitatively controls the extent of damage using a pulling device, which can accurately quantify the damage degree of the optic nerve [10]. The LPON model has achieved pathological changes in RGC survival rate and retinal tissue morphology similar to ONC [10]; it has been applied in traumatic optic neuropathy (TON) experiments [11]. This study aimed to evaluate the effects of varying forces on RGC survival rate and visual conduction function. In addition, we explored the changes in the level of autophagic responses in this model.

## Methods

### Animal experiments

Sixty specific-pathogen-free (SPF) male Wistar rats (7 weeks)weighing 180 ± 20 g were housed in a humidity- and temperature-controlled environment with a 12-h light/dark cycle and freely accessible food and water. Modeling surgery was performed after a 7-day acclimation period.

### Establishing optic nerve traction

All protocols were followed as previously described [10, 11]. Briefly, rats were anesthetized by intraperitoneally injecting 1% sodium pentobarbital (50 mg/kg), and the periocular area was disinfected using iodophor. The skin was incised along the right orbital rim, and the orbital fascia was incised to expose the orbital bone rim. The supraorbital connective tissue and oblique muscle were dissected under a stereo microscope, followed by bluntly dissecting toward the orbital apex until the optic nerve sheath was exposed. The optic nerve sheath was carefully separated to expose the optic nerve, and the optic nerve was wrapped and tied with 6–0 polyester suture of 2 mm behind the eyeball. One end of the suture was fixed, and the other was connected to the transverse tension gauge. The suture perpendicular to the optic nerve was pulled with a tension of 0.1, 0.3, and 0.5 N for 20 s. After surgery, the skin was sutured, erythromycin eye ointment was applied, and the rats were placed on an electric heating pad at 37 °C to recover. In the sham-operated (SH) group, the optic nerve was exposed without stretching. The right eye was used as a surgical eye, and the left eye was used as a control.

### Immunofluorescence protocol

After modeling, on day 7, the eye tissues of the rats were harvested after anesthetizing. After 12 h of fixation in Davidson's fixative, retinas were dissected into flattened whole mounts and then incubated in goat serum blocking solution (Solarbio, SL038, Beijing, China) for 30 min at 37 °C (10% diluted in phosphate-buffered saline (PBS)). Diluted anti-Brn-3a mouse monoclonal antibody (1/100, Brn-3a, ab245230, Abcam, Cambridge, UK) was added dropwise to the tissue and placed in a 4 °C humid box overnight. After washing with PBS, the cyanine 3-labeled secondary antibody (1/300, Servicebio, GB21303, Wuhan, China) was added dropwise to cover the tissue. The retina tissue was incubated for 2 h at 20 °C, and then rinse with PBS and cover with the anti-fade solution. The tissue was observed under a fluorescence microscope. Two high-power fields were randomly selected from each retina section to be tested, and the average number of Brn-3a positive cells was calculated using ImageJ (version 1.8.0).

### Terminal deoxynucleotidyl transferase dUTP nick end labeling (TUNEL)

TUNEL staining of fragmented DNA was detected on whole retinal sections using a TUNEL Apoptosis Detection Kit (YEASEN, 40308ES20, Shanghai, China) following the manufacturer’s instructions. After routine dewaxing and rehydration of the sectioned specimens, proteinase K solution was added dropwise, incubated at room temperature for 20 min, and rinsed with PBS. 1X equilibration buffer (100 μL) was added dropwise, covering the sample area, and incubated at room temperature for 10–30 min. The Alexa Fluor 488-12-dUTP LabelingMix was thawed on ice, and a terminal deoxynucleotidyl transferase (TdT) incubation buffer was prepared (50 μL incubation buffer: 34 μL ddH2O, 10 μL 5 X Equilibration Buffer, 5 μL Alexa Fluor 488-12-DUTP Labeling Mix and 1 μL Recombinant TdT Enzyme). The equilibration buffer was washed off using absorbent paper, and TdT incubation buffer was added. The slides were placed in a humid chamber in the dark and incubated at 37 °C for 60 min. Then, PBS was used for incubating and rinsing. The slides were immersed in a staining jar containing propidium iodide (PI) solution (1 pg/mL, freshly prepared and diluted in PBS) in the dark for 5 min at room temperature. After washing the samples, PBS was added, and the samples were analyzed under a fluorescence microscope. Apoptotic cells on tissue sections showed red fluorescence, and nuclei showed blue fluorescence. The number of apoptotic cells in ganglion cell layer (GCL) was counted using ImageJ (version 1.8.0). Tissues were treated with DNase I for positive control.

### Western blotting

Retinal tissue was extracted and lysed with RIPA (Solarbio, R0010, Beijing, China) lysis buffer for 30 min on ice. After centrifuging the lysate at 12,000 rpm for 15 min at 4 °C, the supernatant was collected. The protein concentration was determined using the BCA protein assay kit (Solarbio, PC0020, Beijing, China). The same amount of protein was loaded on a 10% sodium dodecyl sulfate–polyacrylamide gel electrophoresis (SDS-PAGE) gel. Then, the proteins were transferred to polyvinylidene fluoride (PVDF) membranes. The membranes were immersed in a blocking solution of 50 g L^−1^ nonfat dry milk for 2 h. The corresponding primary antibodies (1/1000, LC3B, 18725-1-AP, Proteintech, USA; 1/1000, P62, AF5384, AffinitY, USA) were added to the membrane overnight at 4 °C. Then, the membranes were incubated with the corresponding secondary antibody (1/1000, MDL, MD912565, Hebei, China) for 1 h at 37 °C on a shaker. Finally, immunoreactive bands were visualized using enhanced chemiluminescence (ECL, Solarbio, PE0010, Beijing, China) and detected using an automated image analysis system (170-8280, ChemiDoc MP Chemiluminescence Imaging System; Bio-Rad, CA, USA). Statistical analysis was performed with β-actin as an internal reference.

### Flash visual evoked potentials (FVEP)

The rats were tested using FVEP 7 d after modeling. After dark adaptation for 12 h, the rats were intraperitoneally injected with 1% pentobarbital as general anesthesia (40 mg/kg). An electrophysiological tester (Roland Electronics Co., Ltd., Keltern, Germany) was used to record the waveforms. A filamentous silver–silver chloride electrode as the working electrode was buried at the midpoint of the connection between the roots of the ears. The reference electrode was placed in the mouth, and the ground electrode was placed subcutaneously in the tail. While recording one eye, the other eye was covered with an opaque eye patch. The light intensity was set to 3.0 CD s m^2^, the stimulation frequency was 1.0 Hz, the waveform analysis time was 250 ms, and the band-pass amplifier recording frequency band was 1–100 Hz. The number of waveform stacking was 100. The first negative peak of the waveform is the N1 wave, the positive peak after the N1 wave is the P1 wave, and the positive and negative peaks after the P1 wave are the N2 wave and P2 wave, respectively [12]. The P1 and P2 wave latency (ms) and amplitude (μv) of rats in each group were compared to assess visual function [13–15].

### Statistics

All data were analyzed using Prism 8.0 (Prism®, GraphPad), and measurement data were expressed as the mean ± standard deviation. Pairwise comparisons were performed using the Student’s *t*-test. Multiple group comparisons were performed using a one-way analysis of variance (ANOVA). Statistical significance was set at *p* < 0.05.

## Results

### Changes in the number of RGCs on day 7 after modeling

**Fig. 1:**
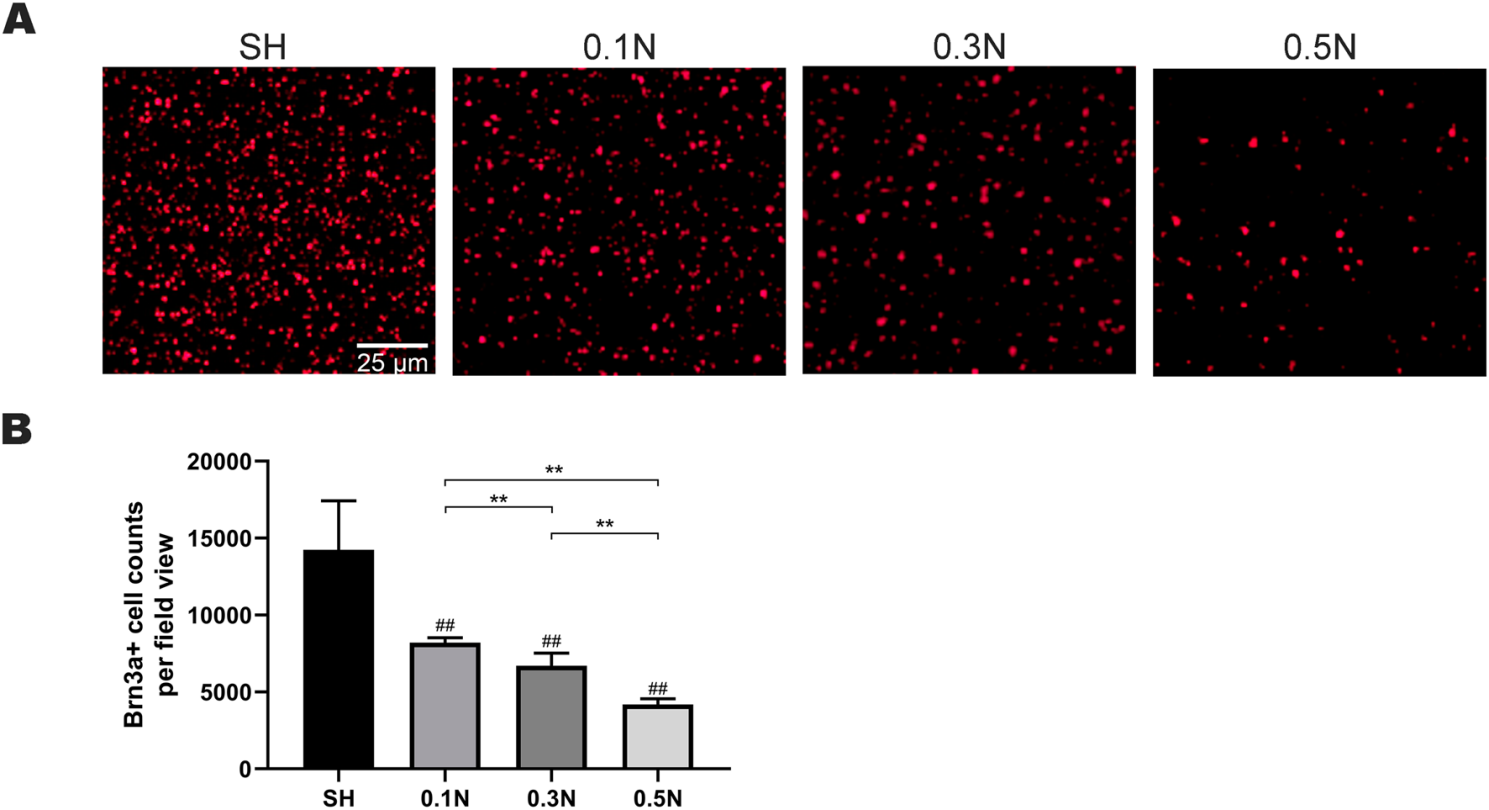
Changes in the number of RGCs on the seventh day after modeling. **a** Immunofluorescence detection of retinal tissue in SH (sham operation group) and model groups (0.1 N/0.3/0.5 N group); **b** Graphic display of the number of Brn3a-labeled RGCs in SH and model group (*n* = 4). Columns, mean ± SE; #*p* < 0.05 or ##*p* < 0.01, compared with the SH group; *p < 0.05 or **p < 0.01, comparison between all model groups

To verify the effect of different forces on the number of RGCs after acting on the optic nerve, immunofluorescence was used to detect the survival of Brn3a-labeled RGCs 7 d after modeling [16]. The number of Brn3a positive cells in the model group was less than that in the SH group. In addition, the number of Brn3a positive cells in the 0.5 N group was less than that in the 0.1 and 0.3 N groups, and those in the 0.3 N group were less than those in the 0.1 N group, with statistically significant differences (*p* < 0.01) (Fig. 1). The results suggest that different forces acting on the optic nerve lead to different numbers of RGCs.

### Changes in the number of apoptotic RGCs in GCL on day 7 after modeling

**Fig. 2:**
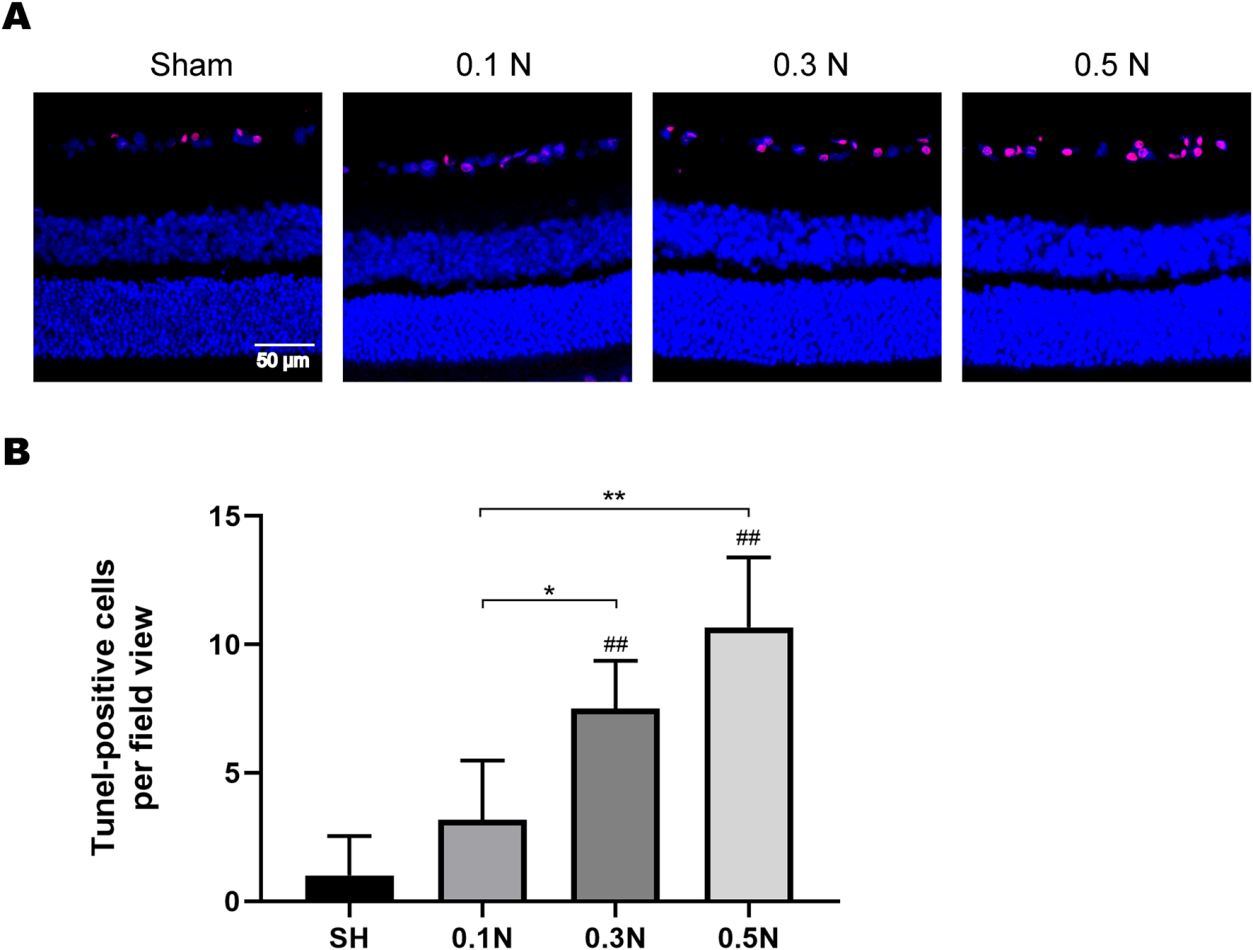
Changes in the number of apoptotic cells in GCL on the seventh day after modeling. **a** TUNEL staining analysis of retinal tissue in SH (sham operation group) and model groups (0.1 N/0.3/0.5 N group); **b** Graphic display of the number of apoptotic cells in SH and model group (*n* = 6). Columns, mean ± SE; #*p* < 0.05 or ##*p* < 0.01, compared with the SH group; *p < 0.05 or **p < 0.01, comparison between all model groups

To further validate our findings, TNNEL staining was used to verify the number of apoptotic cells in GCL in each group on the seventh day after modeling. The number of apoptotic cells of the 0.3 and 0.5 N groups was higher than that of the SH group (*p* < 0.01), whereas that of the 0.1 N group showed no statistical significance. Furthermore, the number of apoptotic cells was higher in the 0.3 and 0.5 N groups than that in the 0.1 N group (*p* < 0.05, *p* < 0.01) (Fig. 2). The above results indicated that the number of apoptotic cells in GCLs after varying forces acted on the optic nerve were different.

### Changes in visual conduction function on days 1, 3, and 7 after modeling

**Fig. 3:**
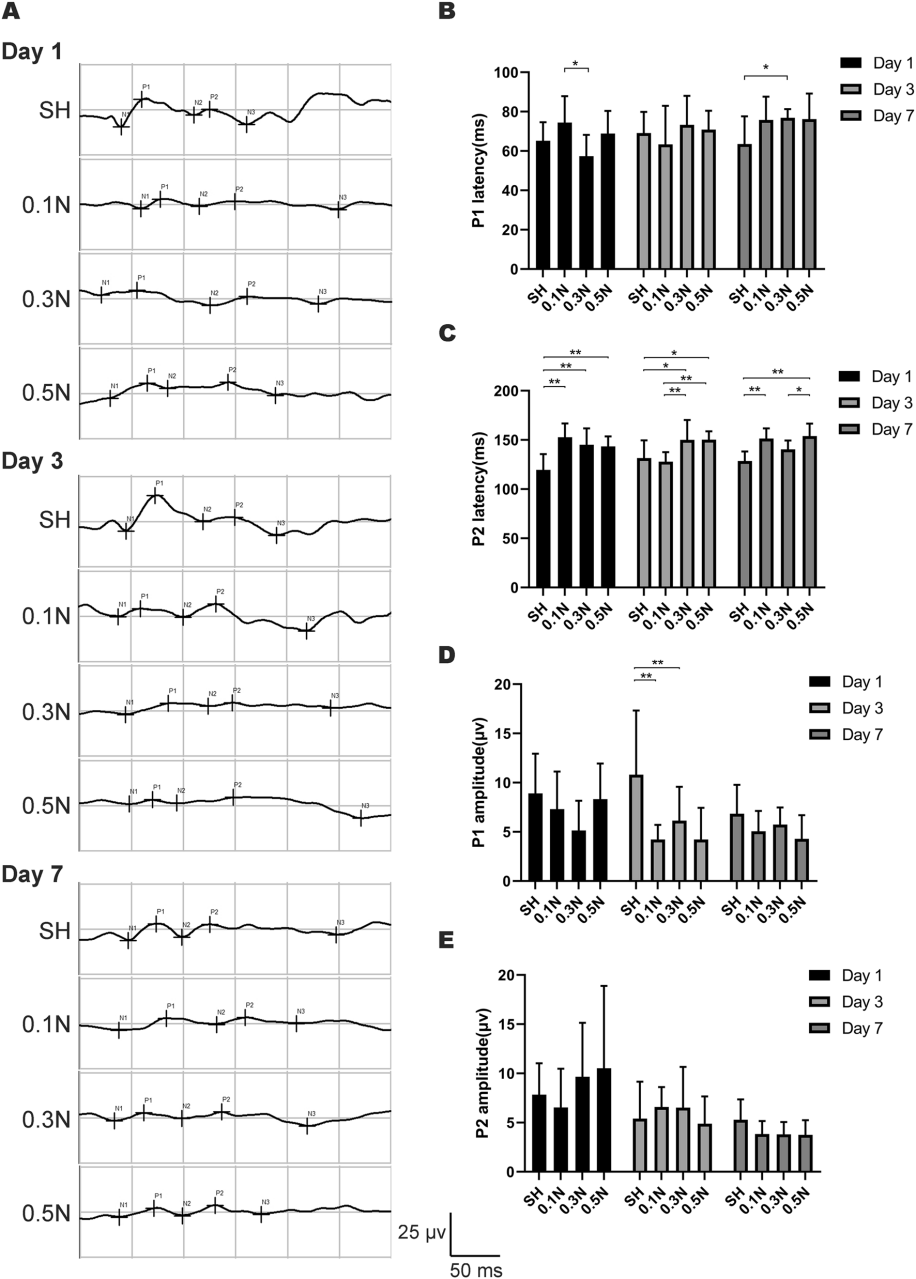
Changes of visual conduction function on day 1/3/7 after modeling. **a** Typical graphs of F-VEP of rats in SH (sham operation group) and model groups (0.1 N/0.3/0.5 N group); **b–c** Graphic display of the P1 (**b**) and P2 (**c**) latency time of F-VEP in each group; **d–e** Graphic display of the P1 (**d**) and P2 (**e**) amplitude of F-VEP in each group (*n* = 9). Mean ± SE; #*p* < 0.05 or ##*p* < 0.01, compared with the SH group; *p < 0.05 or **p < 0.01, comparison between all model groups

To explore the effect of different forces acting on the optic nerve on the electrophysiological function of the optic nerve, FVEP was used to observe optic nerve function on days 1, 3, and 7 after modeling (Fig. 3). In terms of P1 peak latency, there was no statistically significant difference between the model group and SH group on the first day after surgery. Meanwhile, the difference between the 0.1 and 0.3 N groups was statistically significant (*p* < 0.05). There was no significant difference in the P1 peak latency in each group on the third day. On day 7, the difference between the SH group and the 0.3 N group was statistically significant. However, no statistically significant differences were found when SH was compared with 0.1 and 0.3 N groups; in addition, the differences among model groups were not statistically significant (*p* > 0.05).

Regarding the P2 peak latency, the difference between the SH and the model group on day 1 after modeling was statistically significant (all *p* < 0.01), whereas the difference between the model groups with different forces was not statistically significant. On day 3, there was a statistically significant difference in P2 peak latency between the SH group and the 0.3 and 0.5 N groups (*p* < 0.05 and *p* < 0.05, respectively); in addition, compared with the 0.1 N group, the 0.3 and 0.5 N groups showed longer latency, both their differences being statistically significant (*p* < 0.01 and *p* < 0.01, respectively). On day 7, there was a statistically significant difference between the SH group and the 0.1 and 0.5 N groups (*p* < 0.01 and *p* < 0.01, respectively), whereas the difference between SH and 0.3 N group was not statistically significant (*p* > 0.05); in addition, the 0.5 N group showed longer latency than the 0.3 N group (*p* < 0.05).

Regarding the P1 peak amplitude, the SH amplitude on day 3 was higher than that of the 0.1 and 0.3 N groups (*p* < 0.01 and *p* < 0.01, respectively). There was no statistically significant difference in the P1 peak amplitude between the groups on days 1 and 7 after modeling. Although there were differences in the P2 peak amplitude between the groups, the differences were not statistically significant. In general, the above results showed that the difference in the force will lead to the difference in the latency of the P2 peak of F-VEP.

### Changes in autophagy levels on day 7 after modeling

**Fig. 4:**
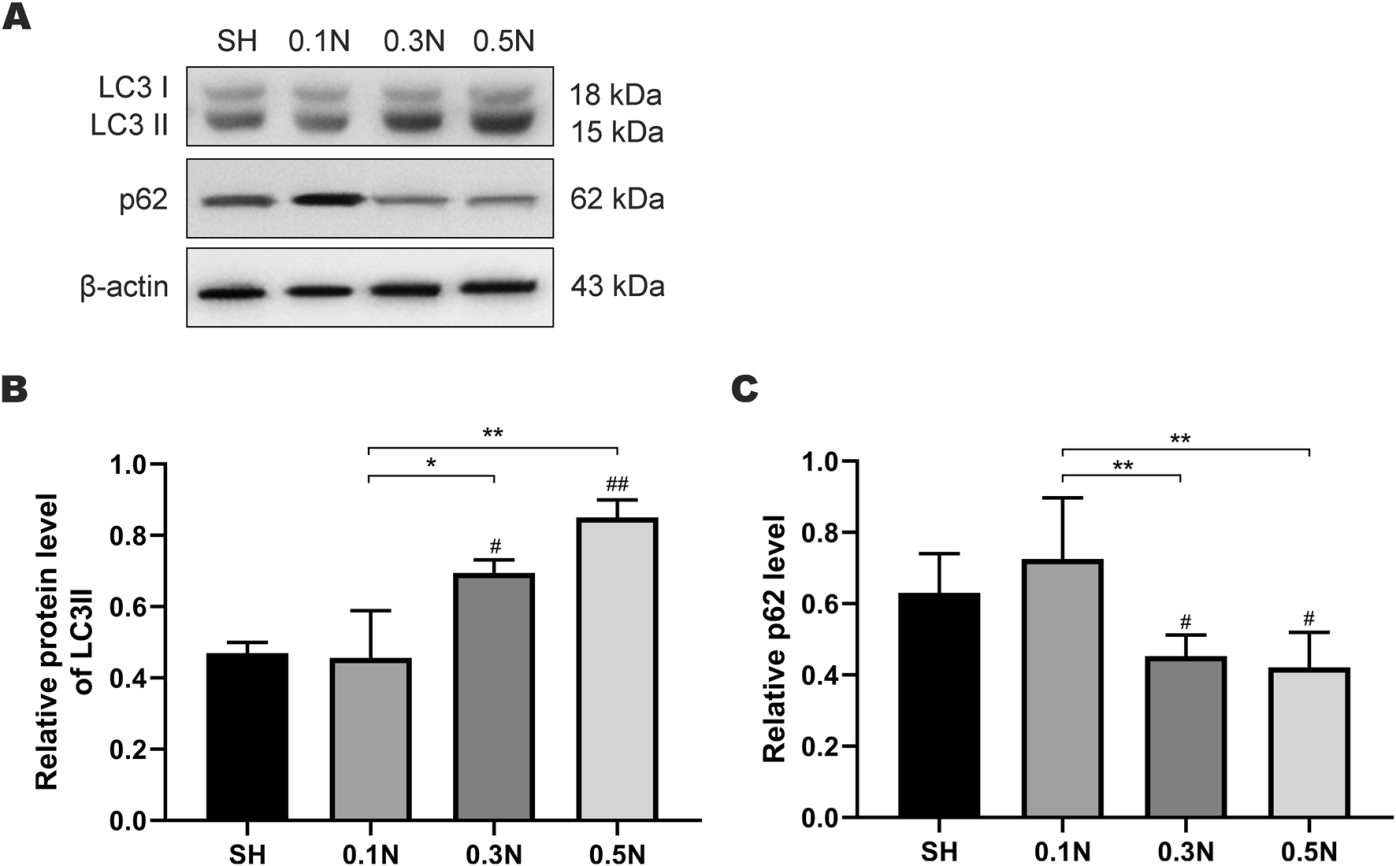
Changes in autophagy levels on day 7 after modeling. **A** Western-Blot was used to detect LC3 and P62 protein level in the retina tissue of rats in SH (sham operation group) and model groups (0.1 N/0.3/0.5 N group); **b–c** Graphic display of LC3 II (**b**) and P62 (**c**) protein level in each group (*n* = 3); Columns, mean ± SE; #*p* < 0.05 or ##*p* < 0.01, compared with the SH group; *p < 0.05 or **p < 0.01, comparison between all model groups

In order to explore the changes of autophagy levels in retinal tissues under varying forces, western blotting was used to detect the level of LC3II and P62 proteins in retinal tissue on day 7 after model establishment. The expression levels of LC3 II in the 0.3 and 0.5 N groups were higher than those in the SH group (*p* < 0.01), whereas the difference between the expression levels in the 0.1 N and SH groups was not statistically significant. In addition, the LC3 II expression levels in the 0.3 and 0.5 N groups were higher than those in the 0.1 N group (*p* < 0.05 and *p* < 0.01, respectively). Furthermore, the expression levels of the P62 protein were detected. The levels in the 0.3 and 0.5 N groups were lower than those in the SH group (*p* < 0.05), whereas the P62 levels in the 0.3 and 0.5 N groups were lower than those in the 0.1 N group (*p* < 0.01). There was no significant difference in the expression levels of LC3 II and P62 proteins in retinal tissue between 0.3 and 0.5 N groups (Fig. 4). The above results showed that the action of different forces on optic nerve would lead to different levels of autophagy in retinal tissues, and the autophagy level also increased with the increase in the force.

## Discussion

For the ONC injury model, the different applied forces lead to different degrees of primary and secondary damage to the optic nerve. However, to our knowledge, few studies have observed the effects of varying forces of injury on the pathological changes of the ONC model. In this experiment, we studied the effects of different pulling forces on the optic nerve injury model using a tensiometer. We observed the RGC number and visual pathway function in model rats under varying forces in addition to the changes in autophagy proteins. The number of survival and apoptosis of RGCs in GCL in the model rats were different from those under varying forces, as were the levels of autophagy proteins. In addition, FVEP revealed that the differences in visual pathway function between the groups were mainly in the P2 peak latency. In conclusion, we observed the differences in the optic nerve injury model under varying forces, specifically in optic neuron loss, conduction function of the optic nerve, and autophagy level.

In an ONC model, the optic nerve is directly crushed using surgical forceps. However, differences in force and durability of surgical instruments can affect the degree of injury. We intended to maintain consistency in optic nerve injury by controlling the force, achieved using a tensiometer. Using the tensiometer to pull the optic nerve after knotting produced an injury similar to that of the ONC injury model while providing relatively precise control over the amount of force applied. The number of RGCs in the model groups was decreased compared with that in the SH group. Moreover, the number of RGCs showed a consequent decrease with increasing force, and the differences in RGC counts between the different force groups were significant.

The pathological changes after optic nerve injury include two pathological stages: primary injury and secondary injury [17, 18]. Mechanical damage, axonal disruption, and impaired blood supply can cause immediate primary damage, resulting in cell death at the injury site. The surrounding area receives various molecular signals and factors that activate biochemical cascades of neuroinflammation, excitotoxicity, and apoptosis, aggravating the lesions of the primary injury [19, 20]. Although the prognosis of optic nerve injury is closely related to the secondary injury, the extent of the primary injury also affects the immediate outcome of the injury [17]. Our results suggest a correlation between RGC survival rate and the degree of injury. Similarly, a previous study reported changes in the number of RGCs when the same force was applied at different times, and the number of RGCs decreased when extending the clamping time of the optic nerve [21].

Apoptosis is involved in secondary degeneration of the RGC after the optic nerve injury [19, 22], which is evidenced by the increased expression of the pro-apoptotic genes Bad and Bax after injury [23] and the increased number of TUNEL staining positive cells [19]. In addition, Hoechst staining [23] and immunohistochemical staining for caspase-3 [24] confirmed RGC apoptosis. Our TUNEL staining results showed the occurrence of apoptosis in GCL one week after injury only in the 0.3 and 0.5 N groups; the 0.1 N group did not show a statistically significant difference compared to the SH group. An increasing apoptosis trend in the GCL with increasing injury force was observed, although no significant difference was observed between the 0.3 and 0.5 N groups. The reason for this trend remains unclear and may be related to oxidative stress [25, 26], autophagy [27], and calcium overload [28]. The production of large amounts of reactive oxygen species (ROS) after axonal injury causes damage to cellular lipids, proteins, and nucleic acids, leading to apoptosis [29, 30]. Calcium accumulation leads to excessive ROS production in mitochondria, aggravating oxidative stress damage [31, 32]. In addition, excessive autophagy leads to RGC death [33, 34].

Autophagy occurs in optic nerve injury models, and their levels increase after injury [35–37]. The LC3 II expression level increases 24 h after injury [34] and reaches the highest on day 7 after clamping injury [35]. In addition, the transcript levels of Atg5 and Atg7, the autophagy-related genes, in RGC soma increased between days 3 and 10 after injury [38]. Similarly, we observed higher LC3 II levels in the 0.3 and 0.5 N model groups than those in the SH group. In addition, LC3 II levels in the 0.3 and 0.5 N groups were higher than those in the 0.1 N group, suggesting that the autophagy level may increase with increasing injury degree. Although the difference between the LC3 II levels in the 0.3 and 0.5 N groups was not statistically significant, we saw this trend in the results: the level of autophagy showed a positive correlation with the damage degree (Fig. 4). In addition, the LC3 II level in the 0.1 N group was not different from that in the SH group, suggesting that a small force may not trigger the autophagic response.

Similar changes occurred in P62 expression levels in the retinal tissue but were not significant. P62 protein can be degraded by autophagic flux. When autophagy is inhibited, P62 levels increase, and a decrease in P62 levels can be observed when autophagy is induced [37, 39]. Consistent with the results for LC3 II, P62 levels were decreased in the 0.3 and 0.5 N model groups; however, no difference was observed between the 0.1 N and the SH group. We noticed an upward trend in autophagy with increasing force in the results, accompanied by a decrease in the number of RGC cells. Although some studies have reported the protective effect of autophagy after optic nerve injury [38–43], excessive autophagy can lead to RGC death [33, 34]; Brn3a immunohistochemistry and TUNEL results seem to reflect this. However, it should be noted that with the aggravation of the degree of injury, the pathological effects caused by mechanical injury, blockage of blood flow supply, as well as secondary inflammation, apoptosis may play a more important role [17, 18]. The protective role of autophagy in this still needs further investigation at different levels of autophagy (Fig. 4).

Optic nerve function is observed using FVEP, which reflects the functional state activity from the retina to the visual cortex. The P2 peak latency appeared more sensitive than the P1 peak latency (Fig. 3). The P1 peak latency was significantly different only between the 0.1 and 0.3 N groups on day 1 and between the SH and 0.3 N groups on day 7. For the P2 peak, the model group showed longer latency than SH on day 1, whereas there was no difference between the model groups. However, on day 3, after modeling, the P2 peak latency in the 0.3 and 0.5 N groups was longer than that in the 0.1 N group. On day 7, the P2 peak latency of the 0.5 N group was longer than that of the 0.3 N group. The FVEP latency can be used to quantify the conduction function of the optic nerve [44–46]. The prolongation of VEP latency is associated with the number of demyelinated nerve fibers [47]. The initial lesion size is the most important factor in determining the outcome of remyelination and continuous axonal loss [48, 49]. In our model, more functional nerve fibers were damaged with increasing damaging force, thereby increasing the number of axonal loss and demyelination. However, no corresponding increase in latency was observed with increasing injury force, which may be due to several reasons. One is that healthy axons in the center play a compensatory function, offsetting the loss of function of damaged axons. Similarly, the damaged axonal function may also be compensated in areas of the cerebral cortex. Autophagy alleviates demyelination and reduces neuronal degeneration [50, 51]. Increased autophagy levels that alleviate demyelination of the optic nerve may also play a role in this process. In addition, the prolongation of latency may not only be related to axonal demyelination but also other factors, such as changes in the local microenvironment and axonal regeneration [52].

The FVEP amplitude reflects the number of functional optic nerve fibers [47, 53, 54], and axonal loss in experimental optic nerve demyelination has a strong linear relationship with reduced amplitude [47]. However, we observed a large variation in the amplitudes of the P1 and P2 peaks. Except for the lower P1 peak amplitude in the 0.1 and 0.3 N groups than that in the SH group on day 3, there was no significant difference in the P1 and P2 peak amplitude among the rats in each group. FVEP detection in rats is affected by individual and perceived factors, such as stimulus design [55, 56], the electrical conductivity of electrodes and underlying tissues [55], cortical anatomy [57], and general levels of cortical activity [58–60]. The measurement results have large variability and volatility [47, 55, 61]. We considered the dark adaptation before the test, the depth of anesthesia, and the implantation position and depth of the test electrodes. For each measurement, we administered repeated external stimulation 100 times to minimize electroencephalograph noise [62]. Only graphs with error waveforms less than 10 times were included, which ensured the accuracy of the results. The amplitude differences between groups gradually narrowed and converged on day 7, which may be due to optic nerve repair; however, a more likely reason is that the resolution of the surrounding tissue edema reduces the compression on the optic nerve, as the functional recovery of the optic nerve is not observed until 10 weeks or more after optic nerve injury [63, 64]. Overall, we found changes in visual electrophysiological function after different forces were applied to the optic nerve. Considering the high variability of FVEP testing and the lack of a linear relationship found between FVEP testing indexes and the magnitude of applied force, FVEP testing is not an ideal test for assessing the extent of optic nerve damage.

Although the LPON model maintains a certain degree of accuracy in optic nerve damage, the model operation is relatively complicated. Sufficient space around the optic nerve is necessary for knotting operation, which requires enough practice to avoid damaging the surrounding blood vessels and excess tissues. The clamping force in the TON model should not be intense, and the time required should not be prolonged to avoid ophthalmic artery damage [1]. Furthermore, if the clamping force is inadequate, the apoptotic and autophagic responses may not be generated.

In conclusion, we observed differences in optic nerve injury models under varying forces, specifically in the loss of optic neurons, the conduction function of the optic nerve, and autophagy level in retinal tissue. Precise clamping of the optic nerve is necessary during the modeling process. The influence of different forces on different indicators may be mistaken for the therapeutic effect of drugs or other interventions, which will misinterpret the research results and lead to wastage of scientific research resources.

## Supplementary Information

Below is the link to the electronic supplementary material.

Supplementary file1 (JPG 157 kb)

Supplementary file2 (JPG 229 kb)

Supplementary file3 (JPG 125 kb)

Supplementary file4 (MP4 699 kb)

Supplementary file5 (MP4 894 kb)

Supplementary file6 (DOCX 276 kb)
