# Supplementary figures and images for: Optic nerve injury models under varying forces
Source: Int Ophthalmol. 2022 Aug 29;43(3):757–69. doi: 10.1007/s10792-022-02476-2 (PMC10042766; doi:10.1007/s10792-022-02476-2)

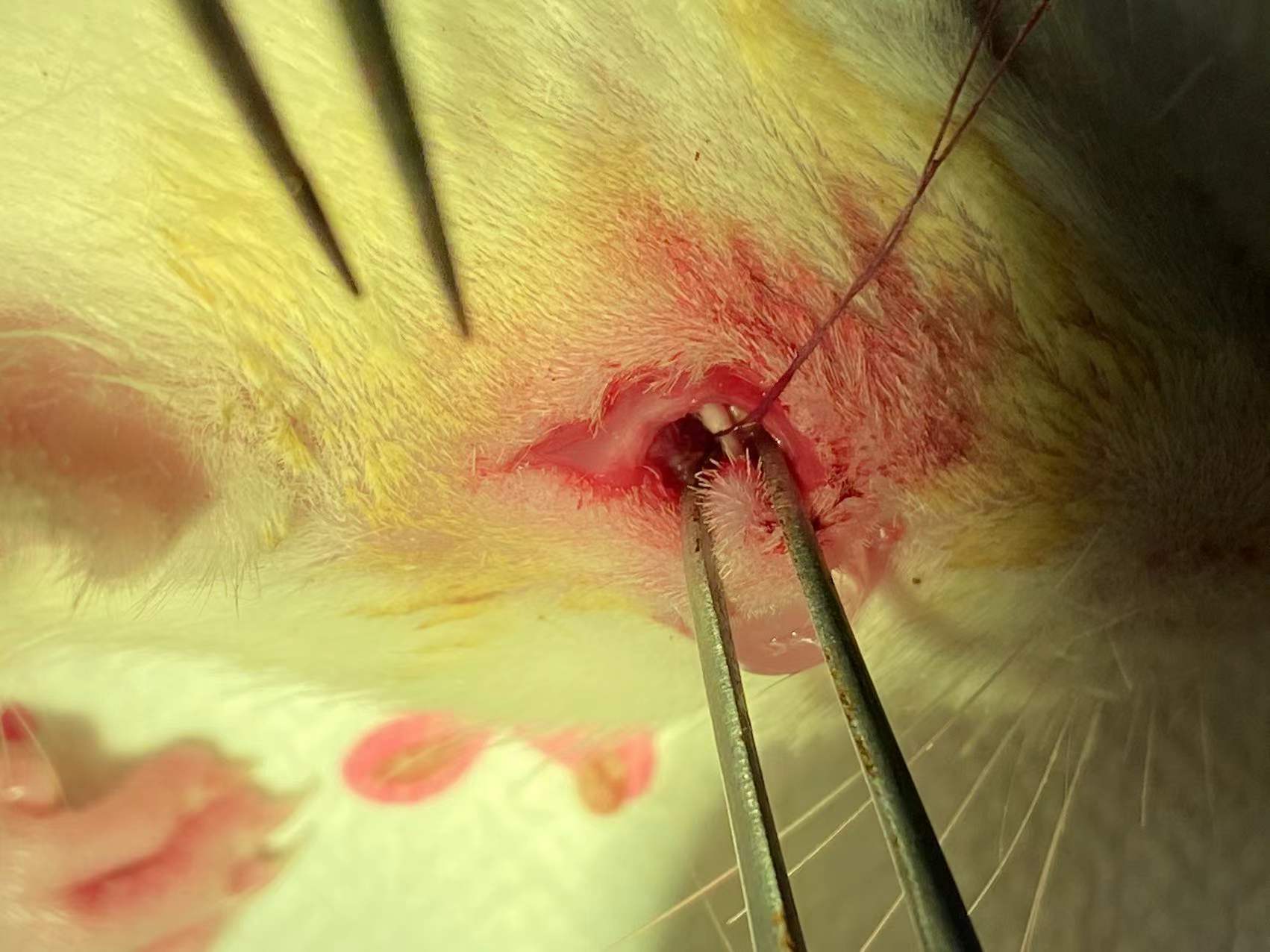

Supplement: Supplementary file 1 — Supplementary file1 (JPG 157 kb) [file 10792_2022_2476_MOESM1_ESM.jpg]

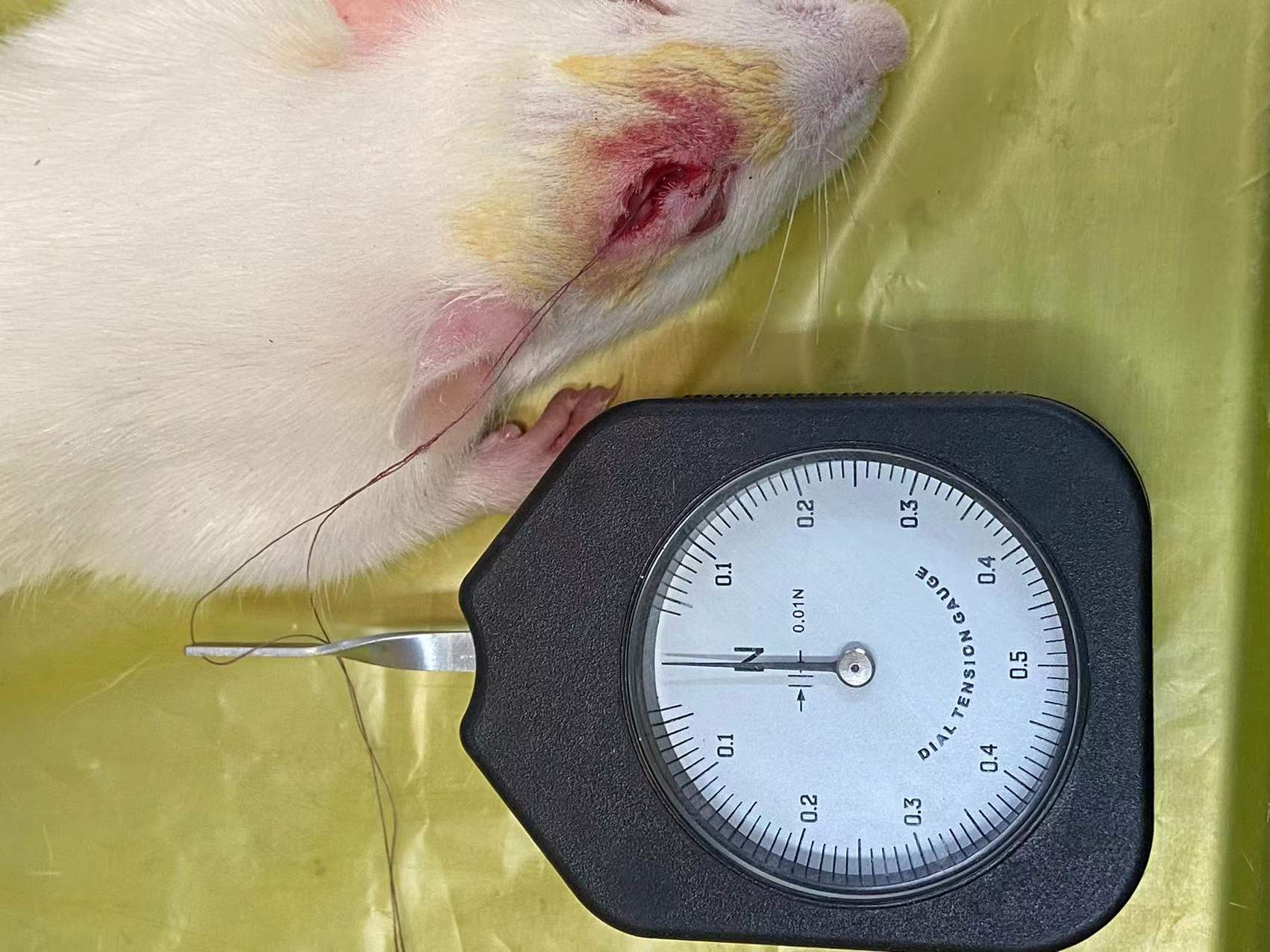

Supplement: Supplementary file 2 — Supplementary file2 (JPG 229 kb) [file 10792_2022_2476_MOESM2_ESM.jpg]

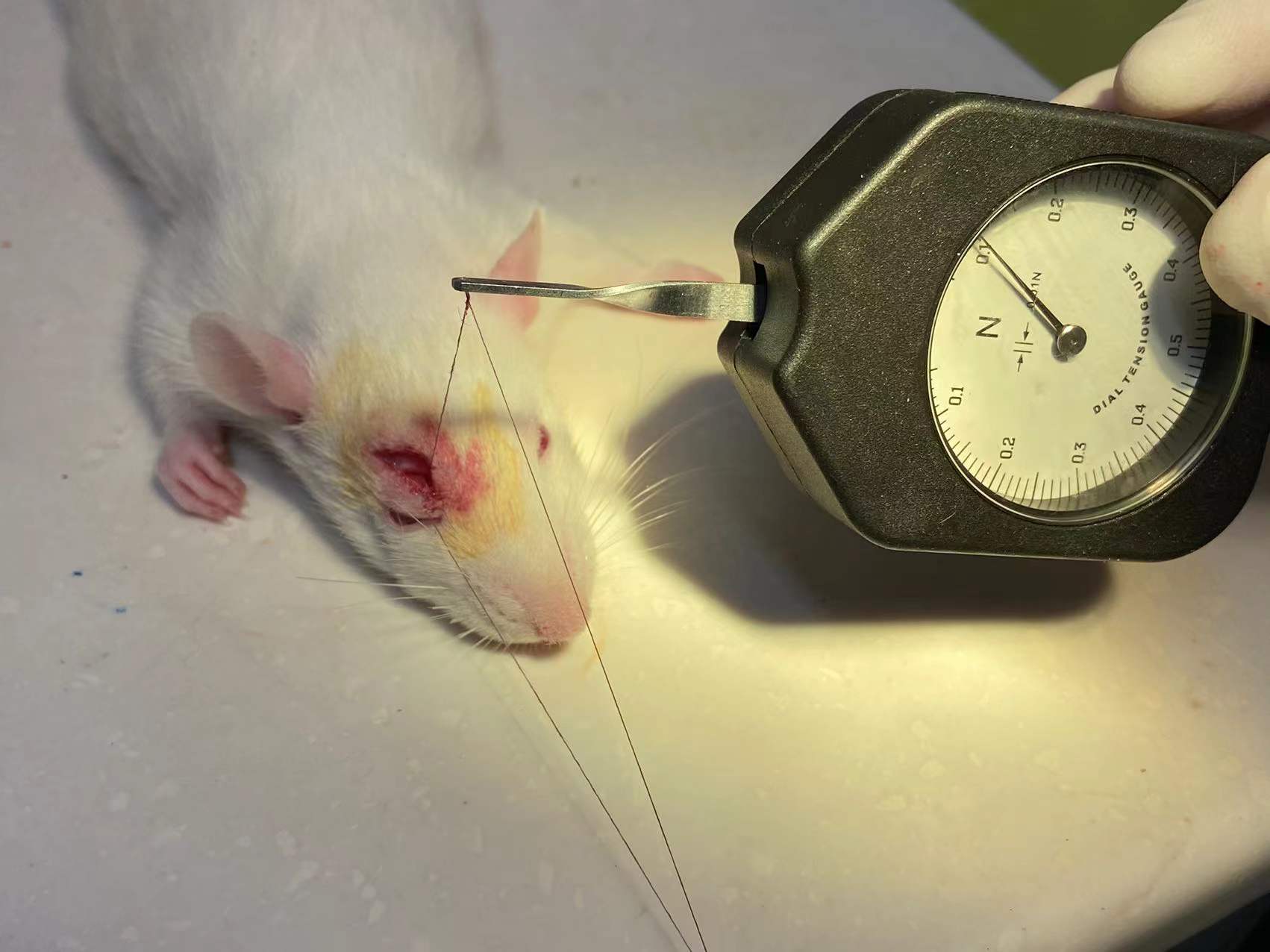

Supplement: Supplementary file 3 — Supplementary file3 (JPG 125 kb) [file 10792_2022_2476_MOESM3_ESM.jpg]

Western-Bolot

LC3


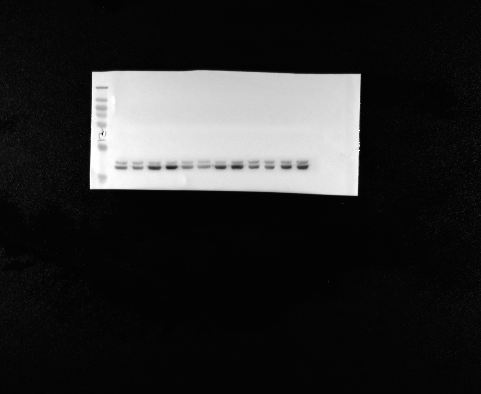


P62


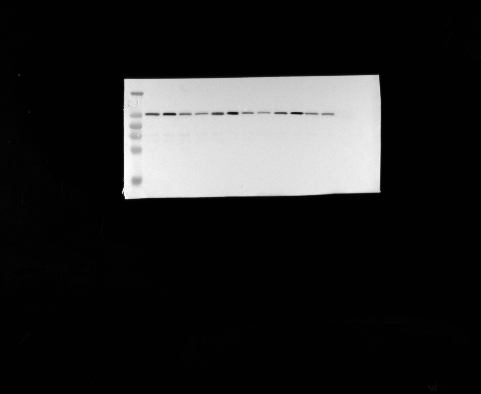


β-actin


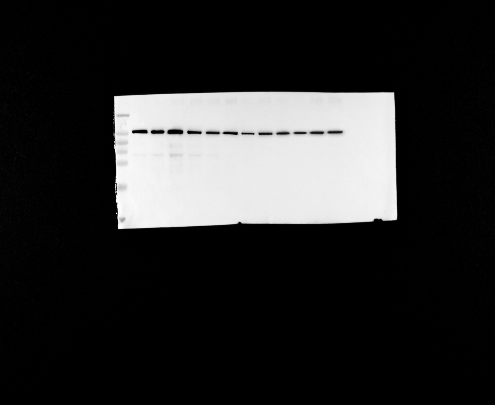

Supplement: Supplementary file 6 — Supplementary file6 (DOCX 276 kb) [file 10792_2022_2476_MOESM6_ESM.docx]
